# Non-parametric combination of multimodal MRI for lesion detection in focal epilepsy

**DOI:** 10.1016/j.nicl.2021.102837

**Published:** 2021-09-25

**Authors:** Jonah Isen, Andrea Perera-Ortega, Sjoerd B Vos, Roman Rodionov, Baris Kanber, Fahmida A Chowdhury, John S Duncan, Parvin Mousavi, Gavin P Winston

**Affiliations:** a School of Computing, Queen’s University, Kingston, Canada; b Centre for Medical Image Computing, University College London, London, UK; c Department of Clinical and Experimental Epilepsy, UCL Queen Square Institute of Neurology, University College London, London, UK; d MRI Unit, Epilepsy Society, Chalfont St Peter, UK; e National Institute for Health Research Biomedical Research Centre at University College London and University College London NHS Foundation Trust, London, UK; f Neuroradiological Academic Unit, UCL Queen Square Institute of Neurology, University College London, London, UK; g Department of Medicine, Division of Neurology & Centre for Neuroscience Studies, Queen's University, Kingston, Canada

**Keywords:** Focal epilepsy, Magnetic resonance imaging, Lesion detection, Voxel-based analysis, Non-parametric combination, MRI-negative

## Abstract

One third of patients with medically refractory focal epilepsy have normal-appearing MRI scans. This poses a problem as identification of the epileptogenic region is required for surgical treatment. This study performs a multimodal voxel-based analysis (VBA) to identify brain abnormalities in MRI-negative focal epilepsy. Data was collected from 69 focal epilepsy patients (42 with discrete lesions on MRI scans, 27 with no visible findings on scans), and 62 healthy controls. MR images comprised T1-weighted, fluid-attenuated inversion recovery (FLAIR), fractional anisotropy (FA) and mean diffusivity (MD) from diffusion tensor imaging, and neurite density index (NDI) from neurite orientation dispersion and density imaging. These multimodal images were coregistered to T1-weighted scans, normalized to a standard space, and smoothed with 8 mm FWHM. Initial analysis performed voxel-wise one-tailed t-tests separately on grey matter concentration (GMC), FLAIR, FA, MD, and NDI, comparing patients with epilepsy to controls. A multimodal non-parametric combination (NPC) analysis was also performed simultaneously on FLAIR, FA, MD, and NDI. Resulting p-maps were family-wise error rate corrected, threshold-free cluster enhanced, and thresholded at p < 0.05. Sensitivity was established through visual comparison of results to manually drawn lesion masks or seizure onset zone (SOZ) from stereoelectroencephalography. A leave-one-out cross-validation with the same analysis protocols was performed on controls to determine specificity. NDI was the best performing individual modality, detecting focal abnormalities in 38% of patients with normal MRI and conclusive SOZ. GMC demonstrated the lowest sensitivity at 19%. NPC provided superior performance to univariate analyses with 50% sensitivity. Specificity in controls ranged between 96 and 100% for all analyses. This study demonstrated the utility of a multimodal VBA utilizing NPC for detecting epileptogenic lesions in MRI-negative focal epilepsy. Future work will apply this approach to datasets from other centres and will experiment with different combinations of MR sequences.

## Highlights

• Multivariate voxel-based analysis useful for lesion detection in focal epilepsy.
• Non-parametric combination algorithm used to combine data from various MR sequences.
• Successful lesion detection demonstrated in MRI-positive and MRI-negative patients.
• Multimodal analysis detected abnormalities from diverse epileptogenic pathologies.
• Sensitivity of multivariate analysis notably higher than univariate analyses.

## 1 Introduction

Focal epilepsy is characterized by recurrent seizures that originate from specific area(s) of the brain (NICE, 2012). Medication is the first line of treatment, but is ineffective in around one third of individuals (Mohanraj and Brodie, 2006). In medically refractory focal epilepsy, surgery may be considered as a therapeutic approach (Moosa and Wyllie, 2013). Although the kind of surgery may vary depending on the individual, successful surgery requires precise spatial identification of the causative brain abnormality. In two thirds of individuals with medically refractory focal epilepsy cases, MRI identifies the brain lesion; however in the remaining one third of cases, MRI scans appear normal (Duncan et al., 2016). If MRI does not identify an epileptogenic lesion, further investigation including invasive stereoelectroencephalography (SEEG) may be required to determine if surgery is viable.

Voxel-based analysis (VBA) is a potential non-invasive method for pre-surgical evaluation. This technique compares brain imaging data on a voxel-wise basis between two groups of subjects, or between a patient and a control group.

Voxel-based morphometry (VBM), a VBA using T1-weighted images, was first introduced by Ashburner and Friston (2000). VBM has been used in various studies to detect abnormalities in focal epilepsy (Bonilha et al., 2004, Martin et al., 2017, Riederer et al., 2008). Martin et al. (2015) reviewed various studies that reported a VBM analysis based on T1 and detailed the general potential of a voxel-based method for lesion detection in focal epilepsy.

Further research has studied the utility of different MRI sequences in a VBA for focal epilepsy. Various works performed a VBA on T2-weighted fluid-attenuated inversion recovery (FLAIR) sequences, and demonstrated FLAIR to be more reliable than T1 in a VBA (Focke et al., 2008a, Focke et al., 2009, Riney et al., 2012). Another study investigated the utility of a VBA on different quantitative MRI contrasts, but yielded results with low specificity (Salmenpera et al., 2007). Other work has explored diffusion tensor imaging (DTI) sequences in VBA methodologies, and has demonstrated the utility of diffusion-weighted images for epileptogenic lesion detection (Focke et al., 2008b, Wang et al., 2018).

A promising approach for lesion detection in focal epilepsy is by performing a VBA on T1-derived contrasts using morphometric analysis program (MAP) (Huppertz et al., 2008, Huppertz et al., 2005). The most commonly analyzed contrast is a junction map, which details the blurring of the grey matter (GM) and white matter (WM) boundary. This modification to the VBA technique has been demonstrated to be a valuable method for detecting subtle MRI abnormalities in epilepsy with high sensitivity (Wang et al., 2015). However, MAP is designed to detect specifically focal cortical dysplasia (FCD) and other cortical malformations, and thus would not be a suitable analysis method for a cohort with a wide range of epileptogenic pathologies.

One commonality among the majority of past studies is that they have focused on individual analyses of differentially processed MRI sequences. A largely unexplored potential improvement for this technique is an analysis of multiple sequences simultaneously. Literature is limited in this field as it requires a large, multimodal dataset.

Non-parametric combination (NPC) is a multivariate analysis technique that has shown the potential for powerful detection of group differences in neuroimaging investigations (Nichols and Holmes, 2001, Winkler et al., 2016). This algorithm provides a technique to perform joint inference on multiple datasets collected from the same subjects, for example considering different MRI modalities jointly. While NPC’s utility in epilepsy focused investigation remains currently unexplored, this technique has been demonstrated to be an effective solution in work focusing on other neurological conditions such as multiple sclerosis and Alzheimer’s disease (Bergsland et al., 2018, Fernandes et al., 2019, Klaassens et al., 2017).

We present a multimodal voxel-based analysis to detect epileptogenic lesions in individuals with refractory focal epilepsy. This approach is first applied to patients who have visible lesions in MRI scans to validate the capabilities of NPC. Then, the analysis is performed on a cohort of patients with focal epilepsy and normal appearing MR images. By performing a NPC analysis on these MRI-negative subjects, we aim to provide a useful non-invasive tool to be utilized in the pre-surgical evaluation of refractory focal epilepsy.

## 2 Methods

### 2.1 Data

This study used data gathered from 69 subjects with medically refractory focal epilepsy undergoing presurgical evaluation at the National Hospital for Neurology and Neurosurgery in London, United Kingdom. The diagnosis of these patients with epilepsy was established through clinical consensus from medical records. These patients with epilepsy fell into two distinct groups. The MRI-positive group was 42 of these individuals who had a visible lesion on MR images. These patients had a range of pathologies, including hippocampal sclerosis (HS) (n = 20), brain tumour (n = 9), FCD (n = 5), cavernoma (n = 3), encephalomalacia (n = 2), ischaemic damage (n = 2), and heterotopia (n = 1). The remaining 27 subjects with epilepsy comprised the MRI-negative group; individuals who had normal appearing MRI scans. 18 of these MRI-negative subjects had ground truth established through stereoelectroencephalography (SEEG), while the remaining 9 subjects had inconclusive SEEG. Additionally, data were collected through the same neuroimaging protocols from 62 healthy control subjects without any history of neurological or psychiatric disease. Further information about these various subjects can be found in Table 1. The use of this data for research was approved by the National Hospital for Neurology and Neurosurgery and the UCL Queen Square Institute of Neurology Joint Ethics Committee, and written informed consent was obtained from all subjects.

**Table 1:** Summary of clinical and demographic information for the MRI-positive, MRI-negative and control cohorts.

	**MRI-Positive Group**	**MRI-Negative Group**	**Control Group**
**Number of Individuals**	42	27	62
**Age** median years [interquartile range]	33.5 [29.25–37.75]	35 [24.5–39]	39 [30–50]
**Gender** male:female	21:21	17:10	22:40
**Age of Onset** median years [interquartile range]	14.5 [6–21.75]	14 [6.5–19]	N/A
**Duration of Disease** median years [interquartile range]	20 [9–28.75]	18 [11–27]	N/A

MRI imaging was performed on a 3 T GE MR750 scanner. Standard imaging gradients were used with a maximum strength of 50 mT/m and a maximum slew rate 200 T/m/s. All of the data were acquired using a body coil for radiofrequency signal transmission and a 32-channel phased array coil for the reception of the signal.

Standard T1-weighted imaging was performed on these subjects with a 1 mm isotropic volumetric three‐dimensional (3D) T1‐weighted inversion‐recovery fast spoiled gradient recalled echo (echo/repetition/inversion time, TE/TR/TI 3.1/7.4/400 ms, field of view (FOV) 224 × 256 × 256 mm, matrix 224 × 256 × 256, parallel imaging acceleration factor 2).

Fluid-attenuated inversion recovery (FLAIR) sequences were performed with fast spin echo, and with variable flip-angle readout (TE/TR/TI: 137/6200/1882 ms, field of view (FOV) 200 × 256 × 256 mm, matrix 200 × 256 × 256).

Multi-shell diffusion MRI data were acquired with a 2 mm isotropic single-shot spin echo sequence that had a FOV of 256 × 256 mm, matrix 128 × 128 and 70 slices (TR/TE = 7600/74.1 ms; ∂/Δ = 21.5/35.9 ms; parallel imaging acceleration factor 2). A total of 115 volumes were acquired with 11, 8, 32, and 64 gradient directions at b-values of 0, 300, 700, and 2500 s/mm^2^ respectively as well as a single b = 0-image with reverse phase-encoding (B0). Distortion correction was performed on this data to allow its use in later registration to T1.

From these diffusion data, the diffusion tensor imaging (DTI) metrics fractional anisotropy (FA) and mean diffusivity (MD) were obtained using REKINDLE in ExploreDTI v4.8.6 (Leemans et al., 2009, Tax et al., 2014). Estimates of intracellular volume fraction as a marker of neurite density index (NDI) were obtained with the NODDI MATLAB Toolbox v0.9 (Zhang et al., 2012). FA, MD and NDI were utilized as previous studies have indicated their promise when used for lesion detection in focal epilepsy (Focke et al., 2008b, Wang et al., 2018, Winston et al., 2014)

Ground truth masks were generated for subjects with epilepsy. In MRI-positive patients, ground truth was represented by lesion masks that were manually drawn by a neurologist (GPW) who has worked extensively in the field of epilepsy neuroimaging research for 15 years. These lesion masks were drawn from visual analysis of coregistered T1 and FLAIR images. As there was no visible lesion in MRI-negative scans, SEEG was utilized to establish ground truth for these subjects. Electrode contacts were automatically extracted from CT scans after electrode insertion and the SEEG data was then reviewed to identify which electrode contacts were involved in seizure onset (SOZ) by the clinical and research team (RR, BK, FAC, JSD). These locations were replaced by a sphere with a radius of 4 mm.

### 2.2 Pre-processing

**Fig. 1:**
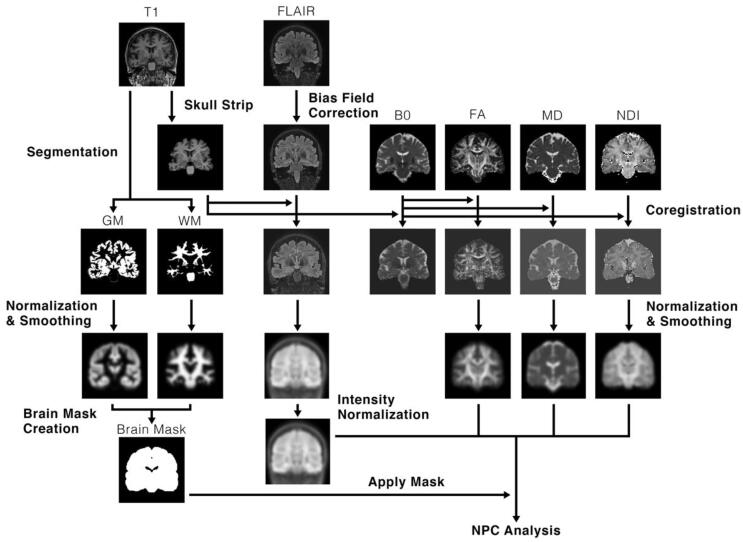
Illustration of the pre-processing workflow performed on the various MRI sequences. T1-weighted images are first skull stripped and segmented into grey matter (GM) and white matter (WM). FLAIR scans are then corrected for bias. FLAIR and the non-diffusion weighted (B0) scan are then coregistered to the skull-stripped T1, and the transformations from the B0 coregistration are applied to FA, MD and NDI. Then, GM, WM, FLAIR, FA, MD and NDI are normalized using DARTEL, and smoothed with 8 mm FWHM gaussian kernel. A brain mask is then created using normalized GMC and WMC. Finally, the mask is applied to the normalized data, and univariate and multimodal analyses are performed on normalized GMC, FLAIR, FA, MD and NDI.

The data were pre-processed (Fig. 1) with in-house MATLAB version R2019b software (Isen, 2021) that utilized subroutines from the Statistical Parametric Mapping 12 (SPM12) suite (Ashburner et al., 2014).

T1-weighted images were segmented into GM and WM maps to be utilized for later normalization and brain mask creation. T1 scans were skull stripped using brain extraction tool (BET) in FSL (Jenkinson et al., 2005) to improve subsequent registration (Fischmeister et al., 2013).

The non-diffusion weighted (B0) images from the diffusion acquisition were rigidly coregistered to the subject’s skull stripped T1 image, and the same transformations were applied to FA, MD and NDI.

Bias field correction was applied to FLAIR scans to account for signal gain variation, and the bias-field corrected FLAIR scans were coregistered to the skull stripped T1 image.

Inter-subject normalization was then performed using diffeomorphic anatomical registration using exponentiated lie algebra (DARTEL) (Ashburner, 2007). This process created an average brain template from every subject’s GM and WM, and also generated flow fields that parameterized the deformations from each subject to this template. Data from each patient were then normalized to MNI space by using these flow fields and average template. Normalization was performed without modulation, approximately preserving per voxel concentration of tissue. The normalized images were smoothed with an 8 mm FWHM gaussian kernel, as per previous voxel-based studies in epilepsy (Beheshti et al., 2018, Focke et al., 2009, Salmenpera et al., 2007).

As FLAIR images are not quantitative like the diffusion metrics, intensity normalization was required in order to make these scans directly comparable between subjects (Focke et al., 2008a). This was performed by segmenting cerebellar WM from normalized FLAIR images by using normalized WM and the probabilistic cerebellar atlas from FSL as a mask (Diedrichsen et al., 2009). The robust mean intensity (5th to 95th percentile) from the segmented cerebellar WM was calculated and the intensity of the overall normalized FLAIR scan was scaled such that the mean cerebellar WM intensity was equal to 1000. The cerebellum was chosen as a reference for intensity normalization as individuals with focal epilepsy are unlikely to have brain abnormalities in the cerebellum.

An average brain mask was created to reduce the potential for false positive findings in non-brain regions. Brain masks were created for each subject by summating normalized grey matter and white matter concentration (GMC and WMC) and excluding voxels with values below 0.5. These individual brain masks were then averaged over all subjects, yielding an average brain mask.

### 2.3 Statistical analysis

Univariate analyses of GMC, FA, MD, NDI and FLAIR were performed with in-house code (Isen, 2021) that used the FMRIB Software Library’s (FSL) randomise tool (Winkler et al., 2014). These individual analyses act as a comparator for the subsequent multimodal NPC analysis.

One-tailed t-tests were performed individually on the normalized, smoothed and masked data, comparing each subject with epilepsy to the control group. This analysis was performed twice per modality, once with a decreased contrast, and then with an increased contrast. Resulting p-values were family-wise error rate (FWER) corrected to correct for multiple comparisons. Threshold-free cluster enhancement (TFCE) was applied with default parameters (height = 2, extent = 0.5, connectivity = 6) (Smith and Nichols, 2009). P-maps were thresholded at a p-value of 0.05, and these thresholded p-maps ultimately represented statistically significant findings from the univariate analyses.

The univariate analyses demonstrated that abnormalities were associated with decreases in FA and NDI, increases in FLAIR and MD, and either increases or decreases in GMC. These same directional changes were observed in previous studies (Focke et al., 2008b, Focke et al., 2009, Riney et al., 2012, Beheshti et al., 2018). Consequently, in order to look for abnormalities in one concordant direction in the simultaneous analysis, normalized FLAIR and MD were multiplied by −1. However, as our NPC analysis looks for changes across multiple modalities with a concordant direction of change, GMC was excluded from the multimodal analysis given the lack of a consistent direction of change.

**Fig. 2:**
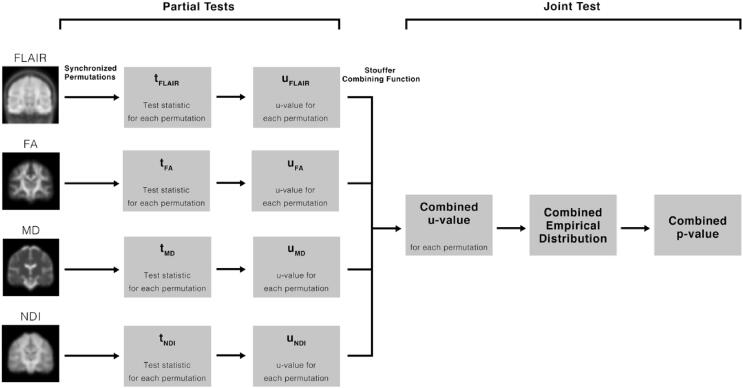
Flowchart detailing the steps of the non-parametric combination algorithm. First, normalized FLAIR, FA, MD and NDI are separately analyzed with 10,000 synchronized permutations. Test statistics are then converted to p-value analogs called u-values. Resulting u-values are then combined with the Stouffer combining function. From the empirical distribution of combined u-values across permutations, a combined p-value is determined.

A voxel-wise, multivariate NPC analysis was then performed (Fig. 2). This algorithm first performs partial tests on each modality, testing each individual hypothesis separately with permutations synchronized across the dataset. Resulting partial test u-values (p-value analogs that act as transitional values) from each permutation are then combined into a joint inference using a combining function. This results in a combined joint statistic for each permutation, and thus a p-value of the joint test from the empirical distribution of combined u-values (Winkler et al., 2016).

This multimodal NPC analysis was performed using FSL’s permutation analysis of linear models (PALM) tool (Winkler et al., 2016). This analysis was performed for each subject with epilepsy in comparison to the control group over the modalities FLAIR, FA, MD, and NDI. NPC was performed with 10,000 permutations. Partial tests were combined into a joint inference using the Stouffer combining function (Winkler et al., 2016). FWER correction and TFCE were applied to resulting p-values across all modalities, and resulting p-maps were thresholded at 0.05.

The control group was analyzed in a leave-one-out cross-validation to assess specificity. Using the same univariate and NPC analysis methods outlined above, each control subject was compared to the control group without that particular subject.

### 2.4 Validation

All metrics to establish the capabilities of this methodology were reported for the univariate tests on GMC, FA, MD, NDI and FLAIR, and for the multimodal NPC analysis. The specificity of this analysis was established from the results of the control group leave-one-out cross validation, and was defined as follows:

specificity = (# of control subjects without findings in p-maps / total # of control subjects) * 100%

**Fig. 3:**
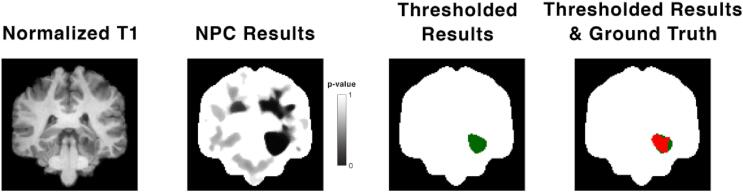
Sample visual comparison between thresholded p-map and ground truth from a MRI-positive group subject with a glioneuronal tumour. The leftmost image shows a T1-weighted scan that is normalized to MNI space and skull-stripped. The second image shows the un-thresholded p-map results from NPC. The third image shows the same results but thresholded at a p-value of 0.05 (green), overlaid on the brain mask. The rightmost image shows the same thresholded results (green) with the ground truth lesion mask overlaid on top (red), again backed by the brain mask. (For interpretation of the references to colour in this figure legend, the reader is referred to the web version of this article.)

The sensitivity of the analysis was established through visual comparison between thresholded p-maps from subjects with epilepsy and their respective ground truth masks (Fig. 3). As 9 MRI-negative subjects had inconclusive ground truth SEEG, sensitivity for this cohort was calculated using only subjects with conclusive SOZ (18/27 subjects). Sensitivity was defined as follows:

MRI-positive sensitivity = (# of p-maps from MRI-positive subjects in visual concordance with lesion masks / total # of MRI-positive subjects) * 100%

MRI-negative sensitivity = (# of p-maps from MRI-negative subjects in visual concordance with SOZ / total # of MRI-negative subjects with conclusive SOZ) * 100%

In MRI-positive patients who have precisely defined ground truth lesion masks, Dice scores were calculated as a quantitative measure of sensitivity. This metric was defined as:

Dice score = (2 * (# of overlapping voxels between MRI-positive subject’s thresholded p-map and lesion mask)) / (# of voxels in thresholded p-map + # of voxels in lesion mask)

Voxel-wise true-positive and false-positive rates were additionally reported, and calculated as follows:

True-positive rate = (Σ (# of voxels in MRI-positive subject’s p-map in concordance with lesion mask) / (# of voxels in MRI-positive subject’s lesion mask)) / (total # of MRI-positive subjects) * 100%

False-positive rate = (Σ (# of voxels in MRI-positive subject’s p-map outside of lesion mask) / (# of voxels in MRI-positive subject’s brain mask)) / (total # of MRI-positive subjects) * 100%

For the MRI-negative group, the SOZ is only localized to specific electrode contact sites so without a precise ground truth lesion definition, dice scores, true-positive rates and false-positive rates cannot be determined.

## 3 Results

**Fig. 4:**
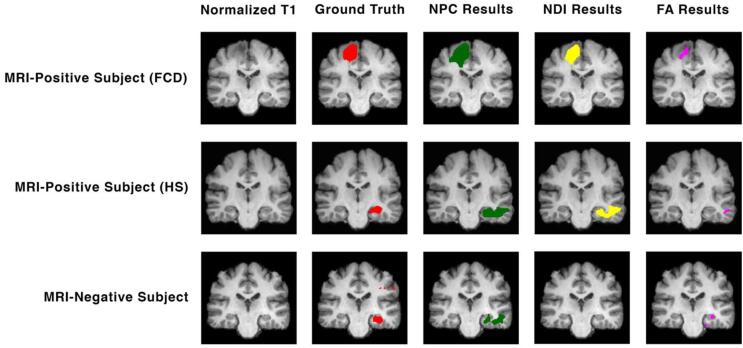
Example lesion detection for 3 different subjects with focal epilepsy. The top row illustrates a MRI-positive subject with FCD, the middle row a MRI-positive subject with HS, and finally the bottom row a MRI-negative subject with normal appearing MR images. The first column of images shows a skull-stripped and normalized T1-weighted scan. The second column shows the ground truth mask (red) overlaid on T1. This ground truth is represented by a lesion mask for the two MRI-positive subjects, and SOZ for the MRI-negative subject. The third column shows the results of the NPC analysis (green) overlaid on T1. The fourth column shows the results of the univariate NDI analysis (yellow) overlaid on T1. The fifth column shows the results of the univariate FA analysis (purple) overlaid on T1. (For interpretation of the references to colour in this figure legend, the reader is referred to the web version of this article.)

For both univariate and multimodal anlayses, the resulting metrics are summarised in Table 2, and example lesion detection from two MRI-positive subjects and one MRI-negative subject are illustrated in Fig. 4.

**Table 2:** Resulting metrics for the univariate and multimodal analyses.

**Result Type**	**Cohort**	**Metric**	**Univariate**						**NPC**
			**Increased GMC**	**Decreased GMC**	**Decreased FA**	**Increased MD**	**Decreased NDI**	**Increased FLAIR**	
**Voxel-Wise**	**MRI-Positive**	**Dice Score**	0.05	0.03	0.08	0.09	0.14	0.10	0.19
		**True-Positive Rate**	3%	2%	7%	8%	20%	7%	41%
		**False-Positive Rate**	0%	0%	1%	1%	2%	0%	6%
**Visual**		**Sensitivity**	29%	26%	38%	48%	62%	50%	81%
	**MRI-Negative**	**Sensitivity**	22%	17%	22%	28%	33%	28%	50%
	**Control**	**Specificity**	98%	97%	97%	100%	97%	98%	97%

### 3.1 Univariate analyses

From the MRI-positive cohort, decreased NDI results were visually concordant with ground truth lesion most often; 26/42 subjects had visually concordant findings, providing a sensitivity of 62%. This was followed in decreasing order by increased FLAIR, increased MD, and decreased FA. GMC yielded the least optimal sensitivity measures at 29% and 26% for increased and decreased GMC, respectively (Table S1). Decreased FLAIR, decreased MD, increased NDI, and increased FA all demonstrated no significant findings in MRI-positive subjects.

Decreased NDI provided the highest dice score of 0.14, while all other univariate analyses resulted in dice scores less than or equal to 0.10 (Table S2). Decreased NDI also provided the highest voxel-wise true-positive measures, detecting on average 20% of lesional voxels in MRI-positive subjects. Increased FLAIR, increased MD, and decreased FA all demonstrated average voxel-wise true-positive rates around 7–8%. Increased and decreased GMC both detected 2–3% of lesional voxels (Table S3). All univariate analyses demonstrated voxel-wise false-positive rates less than or equal to 2% (Table S4).

In patients from the MRI-negative cohort with conclusive ground truth, abnormalities similar to SOZ were observed in 33% of subjects from decreased NDI. Increased FLAIR and MD both yielded sensitivity measures of 28%, while both decreased FA and increased GMC demonstrated 22% MRI-negative group sensitivity. Decreased GMC provided the worst sensitivity at 17% (Table S5). In MRI-negative subjects with inconclusive ground truth, significant findings were found in 1/9 subjects from decreased FA and increased FLAIR analyses, and 2/9 subjects from decreased NDI. Increased GMC, decreased GMC and increased MD analyses provided no significant findings across all MRI-negative, SEEG inconclusive subjects (Table S6).

The leave-one-out cross validation of controls from the unimodal analyses demonstrated minimal false-positive findings in controls and therefore high specificity measures. Decreased FA, decreased NDI and decreased GMC showed significant abnormality detection in 2/62 control subjects, resulting in a specificity of 97%. Increased FLAIR and GMC demonstrated a specificity of 98%, while increased MD had a specificity of 100%.

### 3.2 NPC analysis

In the MRI-positive group, 34/42 subjects’ p-maps from NPC thresholded at a p-value of 0.05 were visually concordant with ground truth lesion masks, resulting in a sensitivity of 81%.

On average, NPC provided a dice score of 0.19, a voxel-wise true-positive rate of 41%, and a false-positive rate of 6% in MRI-positive subjects (Table 3).

**Table 3:** Voxel-wise true-positive lesion detection rates for MRI-positive subjects, grouped by pathology.

**Pathology**	**Univariate**						**NPC**
	**Increased GMC**	**Decreased GMC**	**Decreased FA**	**Increased MD**	**Decreased NDI**	**Increased FLAIR**	
**Tumour** n = 9	0%	1%	0%	2%	30%	13%	51%
**Cavernoma** n = 3	0%	0%	21%	2%	18%	0%	13%
**Encephalomalacia / Ischaemic Damage** n = 4	12%	3%	38%	48%	58%	15%	69%
**Focal Cortical Dysplasia/Heterotopia** n = 6	16%	12%	2%	3%	29%	17%	51%
**Hippocampal Sclerosis** n = 20	0%	1%	4%	5%	6%	1%	31%
**Total MRI-Positive Group** n = 42	3%	2%	7%	8%	20%	7%	41%

In subjects from the MRI-negative cohort with conclusive ground truth, NPC identified abnormalities visually similar to SOZ with a sensitivity of 50% (9/18 subjects). In subjects with inconclusive SEEG, significant findings were observed in 2/9 of subjects (Table S6, Figure S1).

The combined multimodal leave-one-out analysis of controls showed false-positive findings in 2/62 control subjects. This yielded an average specificity from the NPC analysis of 97%.

## 4 Discussion

### 4.1 Key findings

The separate univariate analyses generally provided results with very high specificity, but lacking sensitivity. NDI was the best performing modality, yielding a sensitivity of 62% in the MR-positive group while the analysis of other modalities had sensitivities all below 50%. Analyses on FA, MD and FLAIR all provided sensitivity measures ranging from around 40–50% in MRI-positive subjects, while GMC performed the worst of the individual modalities. The same trends were seen in the MRI-negative cohort analysis; poor sensitivity, NDI outperforming others, and GMC providing the worst results. While the sensitivities observed from these univariate analyses differed, the specificities were comparable between all modalities; near 100% specificity was observed for all individually analyzed modalities.

The simultaneous multmodal analysis using NPC yielded results with generally high specificity and sensitivity. This methodology worked very well on the MRI-positive group; the high visual sensitivity and voxel-wise concordance rate combined with a low voxel-wise false-positive rate demonstrated the potential of a multimodal voxel-based analysis to not only localize lesions reliably, but to also define their shape accurately.

NPC also showed an ability to localize brain abnormalities in MRI-negative patients. While the accuracy of the identified lesion’s shape could not be quantitatively assessed for these subjects due to the nature of the ground truth, findings were visually concordant with SOZ in half of subjects. This finding suggests that NPC in a VBA analysis has potential to detect abnormalities invisible upon review of MR images.

This methodology demonstrated an ability to detect a variety of different pathologies, although at varying degrees of success. MRI-positive subjects with encephalomalacia, ischaemic damage, FCD, and tumours all provided voxel-wise concordant rates above 50%, indicating that the majority of the abnormality was identified. Conversely, the HS and cavernoma cohorts provided less complete lesion detection results, identifying on average 31% and 13% of lesional voxels, respectively.

In the majority (7/9) of MRI-negative subjects who had inconclusive SOZ, the NPC analysis yielded no significant results suggesting that in clinical practice, this technique is unlikely to mislead clinicians. In one subject, significant findings in the left temporal lobe were concordant with interictal epileptiform discharge (IED) masks obtained from SEEG. In the second subject with significant findings, results were not concordant with IED and may be false positive findings.

### 4.2 Multivariate vs. Univariate

In all cases, findings were more abundant from the multimodal analysis than from the univariate analyses; sensitivity was higher for MRI positive and negative subjects, as was the average true-positive rate and dice score from the MRI-positive group. This indicates that combining data from different MRI sequences has the potential to increase the sensitivity of lesion localization in focal epilepsy.

One area where univariate analyses outperformed NPC was with false-positive findings in MRI-positive subjects. Each of the individually analyzed modalities yielded false-positive rates notably lower than the 6% observed from NPC. This is likely attributed to the demonstrated ability for the multimodal approach to detect more subtle change. If the NPC analysis is more sensitive to change and therefore yields more abundant significant findings, then intuitively both true-positive and false-positive values would be increased.

### 4.3 Comparison to previous literature

A previous VBA of FLAIR images demonstrated concordant findings in 14.3% of MRI-negative focal epilepsy patients, and false-positive findings in 4% of controls (Focke et al., 2009). This sensitivity is markedly lower than the 50% observed from our NPC analysis, and is more comparable to the results of the univariate analyses. The specificity of this study is similar to the near 100% observed from our univariate and multivariate analyses. This study differed from ours in various ways; it performed only a univariate analysis on FLAIR, utilized video-EEG telemetry as ground truth, and had a smaller control cohort (n = 25).

Similar comparability to univariate analyses but inferiority to the NPC analysis is demonstrated in a previous study that performed a VBA on different quantitative contrasts (Salmenpera et al., 2007). With these different modalities, 31% of MRI-negative patients had findings in the same lobe as seizure onset, and minimal findings were found in controls.

Kotikalapudi et al. (2018) provided a concordance rate of 46%, and a specificity of 37% from an analysis of differentially processed T1 scans in MRI-negative focal epilepsy. This study yielded a sensitivity higher than our univariate analyses but still lower than the NPC analysis. The specificity of this work was distinctively lower than our analysis. While various MR sequences were used in this work, it was not multimodal in the same nature as our study; the data was not analyzed directly in a multivariate analysis and was only used to preprocess T1-weighted images.

A study by Wang et al. (2015) that utilized MAP in a univariate VBA demonstrated lesion detection with 90% sensitivity, and 67% specificity in MRI-negative patients. This sensitivity measure is significantly higher than that observed from our multivariate NPC analysis (50%), however the specificity of our approach (97%) is noticeably higher than the 67% yielded from the MAP approach. Thus, these two different approaches to subtle lesion detection in focal epilepsy provided promising results in contrasting metrics. While literature has demonstrated that MAP is very useful for abnormality detection in focal epilepsy, MAP is designed to detect only FCD and other cortical malformations. The NPC analysis conversely demonstrated an ability to detect abnormalities across a wide range of pathologies.

A recent study by our group demonstrated a sensitivity of 61% from lesion detection in MRI-negative patients, and a specificity of 99% in controls (Kanber et al., 2021). This sensitivity is comparable to the 50% measure observed with this NPC analysis, as is the specificity compared to the 97% observed in this study. While the dataset is the same as the one utilized in this study, the methodology of the aforementioned study is very different from this work, applying a machine learning approach instead of a purely voxel-based analysis. In our prior work, to train a network to detect lesions reliably, a sufficiently sized training set of subjects with manually drawn lesion masks is required. This is a time-consuming process and is often rate-limiting. In contrast, the present work can be easily performed with an appropriate control set on individual subjects and can thus be more readily applied to data from other centres.

As this study and past literature have used non-identical pre-processing pipelines, various means of establishing sensitivity and specificity, and substantially distinct cohorts of patients, comprehensive conclusions from direct comparisons to past literature should not be made.

## 5 Limitations

This study was limited by the number of available subjects. While the size of the control cohort was comparable to those used in other similar studies (Martin et al., 2017, Bruggemann et al., 2009, Salmenpera et al., 2007), a larger control group may have led to more accurate results. The sample size for MRI-positive subjects with certain pathologies was limited to only a few individuals. The MRI-negative cohort was limited to only 27 subjects as a minority of patients with focal epilepsy have normal appearing MR scans. The resulting metrics from this cohort were additionally limited as we only included patients who had established ground truth in the metric calculations. To obtain a more accurate illustration of the potential of this methodology, future studies should strive to utilize data from a larger cohort.

As p-values may change with the amount of data used in analysis, this form of thresholding may have been a limitation to this study. An alternative method to consider in future studies could be thresholding by effect size; this approach has been demonstrated to provide findings more replicable and consistent than p-value thresholds, regardless of sample size (Vandekar and Stephens, 2021).

The inability to include T1 GMC in the multivariate analysis is a limitation, given the utility of this modality for focal epilepsy lesion detection demonstrated by past VBM analyses (Bonilha et al., 2004, Martin et al., 2017). However, the poor individual performance of GMC from the univariate analysis suggests that it may not have been helpful to improve the results of the NPC analysis. Consideration could instead be given to incorporating T1-derived contrasts from MAP as additional modalities in a multivariate analysis, as previous studies have shown MAP to be a practical tool for subtle abnormality detection in focal epilepsy (Huppertz et al., 2008, Wang et al., 2015).

As results from the MRI-positive group were validated against ground truth lesion masks, a limitation may have come from the quality of these manually drawn masks at representing the full extent of ground truth. Upon additional reviews of the T1 scans subsequent to the NPC analysis, there were a few cases where lesion masks failed to detail subtle features of abnormalities that the NPC analysis did in fact detect. In these cases, voxels were labelled as false-positive when they were in actuality true-positive.

The demonstrated ability for this methodology to detect brain abnormalities not seen in MR images may have also misleadingly inflated false-positive rates in MRI-positive subjects. Lesion masks simply represent the lesional area visible in MR images, but may not in every case represent the full scope of the epileptogenic lesion. In some subjects with HS, NPC detected additional abnormalities in the temporal lobe that were not visible in MR scans. This is interesting, as HS is often associated with temporal pole abnormalities (Winston et al., 2020).

Due to these limitations with the ground truth for the MRI-positive cohort, in many cases voxel-wise false-positive rates were likely inflated and consequently true-positive rates deflated. As such, these voxel-wise metrics should only be perceived as an indicator of this methodology’s capability to detect subtle features of lesions, and not as an exact measure of its success. The visual sensitivity metric is thus a more general and reliable representation of the capability for NPC with multimodal data to detect epileptogenic lesions.

## 6 Conclusion

This work successfully demonstrates the superior performance of a simultaneous, multimodal VBA utilizing NPC for detecting epileptogenic lesions in focal epilepsy, both in cases of visible and non-visible lesions in MRI scans. In subjects with MRI-negative focal epilepsy, this may prove especially helpful in the pre-surgical evaluation process for these individuals.

Future work will look to apply this approach to data acquired from other centres to ascertain its capabilities on a broad range of data. Utilizing a larger dataset will improve the reliability and possibly the accuracy of results. Furthermore, future studies will look at different combinations of MRI sequences in a NPC analysis to further improve this methodology.

## CRediT authorship contribution statement

**Jonah Isen:** Conceptualization, Methodology, Software, Validation, Formal analysis, Investigation, Writing - original draft, Writing - review & editing. **Andrea Perera-Ortega:** Methodology, Software. **Sjoerd B Vos:** Methodology, Resources, Data curation, Writing - review & editing. **Roman Rodionov:** Data curation. **Baris Kanber:** Data curation, Writing - review & editing. **Fahmida A Chowdhury:** Data curation. **John S Duncan:** Data curation, Writing - review & editing. **Parvin Mousavi:** Conceptualization, Writing - review & editing, Supervision. **Gavin P Winston:** Conceptualization, Software, Resources, Data curation, Writing - review & editing, Supervision, Project administration, Funding acquisition.

## Declaration of Competing Interest

The authors declare that they have no known competing financial interests or personal relationships that could have appeared to influence the work reported in this paper.
