# Supplementary material for: Non-parametric combination of multimodal MRI for lesion detection in focal epilepsy
Source: Neuroimage Clin. 2021 Sep 25;32:102837. doi: 10.1016/j.nicl.2021.102837 (PMC8503566; doi:10.1016/j.nicl.2021.102837)
Supplement: Supplementary data 1 [file mmc1.docx]

**Supplementary Table 1.** Subject-specific visual concordances with ground truth from MRI-positive cohort

| **Subject** | **Visual Concordance with Ground Truth** | | | | | | |
| --- | --- | --- | --- | --- | --- | --- | --- |
|  | **Increased GMC** | **Decreased GMC** | **Decreased FA** | **Increased MD** | **Decreased NDI** | **Increased FLAIR** | **NPC** |
| **1** | N | N | Y | N | Y | Y | Y |
| **2** | Y | N | N | Y | Y | Y | Y |
| **3** | N | N | Y | N | Y | Y | Y |
| **4** | N | N | N | N | N | N | N |
| **5** | Y | N | Y | Y | N | Y | Y |
| **6** | N | N | N | N | N | N | N |
| **7** | N | N | Y | Y | Y | Y | Y |
| **8** | N | N | N | Y | Y | Y | Y |
| **9** | N | N | N | N | N | Y | Y |
| **10** | N | N | N | N | Y | Y | Y |
| **11** | Y | Y | Y | Y | Y | Y | Y |
| **12** | N | N | N | N | N | N | N |
| **13** | N | N | N | N | Y | Y | Y |
| **14** | N | N | N | N | Y | Y | Y |
| **15** | Y | N | Y | Y | Y | N | Y |
| **16** | N | N | N | N | N | N | N |
| **17** | N | N | N | N | N | N | N |
| **18** | N | Y | N | Y | N | Y | Y |
| **19** | Y | N | Y | Y | Y | Y | Y |
| **20** | N | N | Y | Y | N | Y | Y |
| **21** | N | N | N | N | Y | N | Y |
| **22** | Y | Y | Y | Y | Y | Y | Y |
| **23** | N | Y | N | Y | N | N | Y |
| **24** | N | N | Y | Y | Y | N | Y |
| **25** | N | Y | N | N | Y | Y | Y |
| **26** | N | N | N | Y | Y | N | Y |
| **27** | Y | N | N | N | N | N | N |
| **28** | Y | N | Y | Y | Y | Y | Y |
| **29** | N | N | N | N | N | N | N |
| **30** | N | Y | Y | Y | Y | N | Y |
| **31** | N | Y | Y | Y | Y | Y | Y |
| **32** | Y | N | N | N | Y | N | Y |
| **33** | N | N | N | N | Y | N | Y |
| **34** | Y | Y | Y | Y | N | N | Y |
| **35** | N | N | N | N | N | N | N |
| **36** | Y | Y | N | N | N | Y | Y |
| **37** | N | N | N | N | Y | N | Y |
| **38** | N | N | Y | Y | Y | N | Y |
| **39** | N | N | Y | Y | Y | N | Y |
| **40** | N | Y | N | N | N | Y | Y |
| **41** | Y | N | N | N | Y | Y | Y |
| **42** | N | Y | N | Y | Y | N | Y |
| **Total MRI-Positive Group** | 12/42  (29%) | 11/42  (26%) | 16/42  (38%) | 20/42 (48%) | 26/42  (62%) | 21/42 (50%) | 34/42  (81%) |

**Supplementary Table 2.** Subject-specific Dice scores from MRI-positive cohort

| **Subject** | **Dice Score** | | | | | | |
| --- | --- | --- | --- | --- | --- | --- | --- |
|  | **Increased GMC** | **Decreased GMC** | **Decreased FA** | **Increased MD** | **Decreased NDI** | **Increased FLAIR** | **NPC** |
| **1** | 0 | 0 | 0.1 | 0 | 0.07 | 0.21 | 0.03 |
| **2** | 0.13 | 0 | 0 | 0.06 | 0.04 | 0.28 | 0.48 |
| **3** | 0 | 0 | 0.33 | 0 | 0.04 | 0.47 | 0.12 |
| **4** | 0 | 0 | 0 | 0 | 0 | 0 | 0 |
| **5** | 0.04 | 0 | 0.21 | 0.07 | 0 | 0.2 | 0.03 |
| **6** | 0 | 0 | 0 | 0 | 0 | 0 | 0 |
| **7** | 0 | 0 | 0.04 | 0.18 | 0.56 | 0.36 | 0.73 |
| **8** | 0 | 0 | 0 | 0.04 | 0.04 | 0.15 | 0.02 |
| **9** | 0 | 0 | 0 | 0 | 0 | 0.11 | 0.15 |
| **10** | 0 | 0 | 0 | 0 | 0.06 | 0.04 | 0.07 |
| **11** | 0.08 | 0.17 | 0.04 | 0.57 | 0.37 | 0.31 | 0.12 |
| **12** | 0 | 0 | 0 | 0 | 0 | 0 | 0 |
| **13** | 0 | 0 | 0 | 0 | 0.1 | 0.08 | 0.04 |
| **14** | 0 | 0 | 0 | 0 | 0.04 | 0.03 | 0.16 |
| **15** | 0.08 | 0 | 0.12 | 0.25 | 0.21 | 0 | 0.26 |
| **16** | 0 | 0 | 0 | 0 | 0 | 0 | 0 |
| **17** | 0 | 0 | 0 | 0 | 0 | 0 | 0 |
| **18** | 0 | 0.02 | 0 | 0.06 | 0 | 0.29 | 0.14 |
| **19** | 0.42 | 0 | 0.22 | 0.48 | 0.4 | 0.16 | 0.45 |
| **20** | 0 | 0 | 0.13 | 0.11 | 0 | 0.08 | 0.24 |
| **21** | 0 | 0 | 0 | 0 | 0.07 | 0 | 0.01 |
| **22** | 0.15 | 0.06 | 0.44 | 0.37 | 0.5 | 0.2 | 0.52 |
| **23** | 0 | 0.02 | 0 | 0.01 | 0 | 0 | 0.17 |
| **24** | 0 | 0 | 0.28 | 0.08 | 0.54 | 0 | 0.21 |
| **25** | 0 | 0.11 | 0 | 0 | 0.2 | 0.04 | 0.86 |
| **26** | 0 | 0 | 0 | 0.1 | 0.71 | 0 | 0.02 |
| **27** | 0.02 | 0 | 0 | 0 | 0 | 0 | 0 |
| **28** | 0.58 | 0 | 0.64 | 0.32 | 0.75 | 0.7 | 0.79 |
| **29** | 0 | 0 | 0 | 0 | 0 | 0 | 0 |
| **30** | 0 | 0.06 | 0.41 | 0.44 | 0.36 | 0 | 0.19 |
| **31** | 0 | 0.11 | 0.15 | 0.13 | 0.1 | 0.12 | 0.24 |
| **32** | 0.04 | 0 | 0 | 0 | 0.06 | 0 | 0.06 |
| **33** | 0 | 0 | 0 | 0 | 0.22 | 0 | 0.11 |
| **34** | 0.18 | 0.06 | 0.18 | 0.1 | 0 | 0 | 0.3 |
| **35** | 0 | 0 | 0 | 0 | 0 | 0 | 0 |
| **36** | 0.23 | 0.02 | 0 | 0 | 0 | 0.11 | 0.08 |
| **37** | 0 | 0 | 0 | 0 | 0.2 | 0 | 0.04 |
| **38** | 0 | 0 | 0.06 | 0.1 | 0.08 | 0 | 0.5 |
| **39** | 0 | 0 | 0.05 | 0.4 | 0.17 | 0 | 0.4 |
| **40** | 0 | 0.04 | 0 | 0 | 0 | 0.04 | 0.17 |
| **41** | 0.06 | 0 | 0 | 0 | 0.02 | 0.1 | 0.01 |
| **42** | 0 | 0.47 | 0 | 0.04 | 0.06 | 0 | 0.11 |
| **Total MRI-Positive Group** | 0.05 | 0.03 | 0.08 | 0.09 | 0.14 | 0.1 | 0.19 |

**Supplementary Table 3.** Subject-specific voxel-wise true positive rates from MRI-positive cohort

| **Subject** | **Voxel-Wise True Positive Rate** | | | | | | |
| --- | --- | --- | --- | --- | --- | --- | --- |
|  | **Increased GMC** | **Decreased GMC** | **Decreased FA** | **Increased MD** | **Decreased NDI** | **Increased FLAIR** | **NPC** |
| **1** | 0% | 0% | 5% | 0% | 33% | 12% | 63% |
| **2** | 7% | 0% | 0% | 3% | 2% | 16% | 32% |
| **3** | 0% | 0% | 20% | 0% | 9% | 31% | 83% |
| **4** | 0% | 0% | 0% | 0% | 0% | 0% | 0% |
| **5** | 2% | 0% | 12% | 18% | 0% | 11% | 95% |
| **6** | 0% | 0% | 0% | 0% | 0% | 0% | 0% |
| **7** | 0% | 0% | 2% | 10% | 69% | 34% | 73% |
| **8** | 0% | 0% | 0% | 2% | 2% | 8% | 38% |
| **9** | 0% | 0% | 0% | 0% | 0% | 6% | 13% |
| **10** | 0% | 0% | 0% | 0% | 3% | 2% | 21% |
| **11** | 4% | 9% | 2% | 42% | 33% | 18% | 51% |
| **12** | 0% | 0% | 0% | 0% | 0% | 0% | 0% |
| **13** | 0% | 0% | 0% | 0% | 5% | 4% | 28% |
| **14** | 0% | 0% | 0% | 0% | 7% | 2% | 44% |
| **15** | 4% | 0% | 31% | 14% | 79% | 0% | 94% |
| **16** | 0% | 0% | 0% | 0% | 0% | 0% | 0% |
| **17** | 0% | 0% | 0% | 0% | 0% | 0% | 0% |
| **18** | 0% | 1% | 0% | 3% | 0% | 17% | 40% |
| **19** | 37% | 0% | 42% | 45% | 88% | 12% | 90% |
| **20** | 0% | 0% | 7% | 16% | 0% | 4% | 64% |
| **21** | 0% | 0% | 0% | 0% | 92% | 0% | 33% |
| **22** | 8% | 3% | 34% | 23% | 60% | 11% | 63% |
| **23** | 0% | 1% | 0% | 2% | 0% | 0% | 43% |
| **24** | 0% | 0% | 16% | 4% | 72% | 0% | 87% |
| **25** | 0% | 6% | 0% | 0% | 11% | 22% | 76% |
| **26** | 0% | 0% | 0% | 5% | 55% | 0% | 30% |
| **27** | 1% | 0% | 0% | 0% | 0% | 0% | 0% |
| **28** | 52% | 0% | 47% | 19% | 60% | 54% | 82% |
| **29** | 0% | 0% | 0% | 0% | 0% | 0% | 0% |
| **30** | 0% | 3% | 38% | 42% | 33% | 0% | 70% |
| **31** | 0% | 13% | 35% | 31% | 29% | 23% | 83% |
| **32** | 2% | 0% | 0% | 0% | 3% | 0% | 14% |
| **33** | 0% | 0% | 0% | 0% | 51% | 0% | 53% |
| **34** | 10% | 3% | 14% | 5% | 0% | 0% | 85% |
| **35** | 0% | 0% | 0% | 0% | 0% | 0% | 0% |
| **36** | 13% | 1% | 0% | 0% | 0% | 6% | 28% |
| **37** | 0% | 0% | 0% | 0% | 11% | 0% | 13% |
| **38** | 0% | 0% | 3% | 5% | 4% | 0% | 51% |
| **39** | 0% | 0% | 4% | 52% | 17% | 0% | 65% |
| **40** | 0% | 2% | 0% | 0% | 0% | 2% | 9% |
| **41** | 3% | 0% | 0% | 0% | 7% | 5% | 15% |
| **42** | 0% | 31% | 0% | 2% | 3% | 0% | 6% |
| **Total MRI-Positive Group** | 3% | 2% | 7% | 8% | 20% | 7% | 41% |

**Supplementary Table 4.** Subject-specific voxel-wise false positive rates from MRI-positive cohort

| **Subject** | **Voxel-Wise False Positive Rate** | | | | | | |
| --- | --- | --- | --- | --- | --- | --- | --- |
|  | **Increased GMC** | **Decreased GMC** | **Decreased FA** | **Increased MD** | **Decreased NDI** | **Increased FLAIR** | **NPC** |
| **1** | 0% | 0% | 0% | 0% | 3% | 0% | 17% |
| **2** | 0% | 0% | 0% | 0% | 0% | 0% | 0% |
| **3** | 0% | 1% | 0% | 0% | 1% | 0% | 4% |
| **4** | 0% | 0% | 0% | 0% | 0% | 0% | 0% |
| **5** | 0% | 0% | 0% | 1% | 0% | 0% | 15% |
| **6** | 0% | 0% | 0% | 0% | 0% | 0% | 0% |
| **7** | 0% | 0% | 0% | 0% | 3% | 2% | 1% |
| **8** | 0% | 0% | 0% | 0% | 0% | 0% | 11% |
| **9** | 0% | 0% | 0% | 0% | 0% | 0% | 2% |
| **10** | 0% | 0% | 0% | 0% | 0% | 0% | 3% |
| **11** | 0% | 0% | 0% | 1% | 1% | 0% | 15% |
| **12** | 0% | 0% | 0% | 0% | 0% | 0% | 14% |
| **13** | 0% | 0% | 0% | 0% | 0% | 0% | 3% |
| **14** | 0% | 1% | 0% | 0% | 4% | 1% | 8% |
| **15** | 0% | 0% | 9% | 0% | 14% | 0% | 13% |
| **16** | 0% | 0% | 0% | 0% | 0% | 0% | 0% |
| **17** | 0% | 0% | 0% | 0% | 0% | 0% | 0% |
| **18** | 0% | 0% | 0% | 0% | 0% | 0% | 2% |
| **19** | 1% | 0% | 6% | 1% | 6% | 1% | 5% |
| **20** | 0% | 0% | 0% | 0% | 0% | 0% | 3% |
| **21** | 0% | 0% | 0% | 0% | 5% | 0% | 14% |
| **22** | 0% | 0% | 1% | 0% | 4% | 0% | 4% |
| **23** | 0% | 0% | 1% | 5% | 3% | 0% | 8% |
| **24** | 0% | 0% | 0% | 0% | 2% | 0% | 14% |
| **25** | 0% | 0% | 0% | 0% | 0% | 2% | 0% |
| **26** | 0% | 0% | 0% | 0% | 0% | 0% | 7% |
| **27** | 0% | 0% | 0% | 0% | 0% | 0% | 0% |
| **28** | 1% | 0% | 0% | 0% | 0% | 0% | 1% |
| **29** | 0% | 0% | 0% | 0% | 0% | 0% | 4% |
| **30** | 0% | 0% | 1% | 1% | 1% | 0% | 12% |
| **31** | 0% | 3% | 8% | 8% | 10% | 6% | 12% |
| **32** | 0% | 0% | 0% | 0% | 0% | 0% | 1% |
| **33** | 0% | 0% | 0% | 0% | 7% | 0% | 18% |
| **34** | 0% | 0% | 1% | 0% | 0% | 0% | 9% |
| **35** | 0% | 0% | 0% | 0% | 0% | 1% | 0% |
| **36** | 0% | 0% | 0% | 0% | 0% | 0% | 3% |
| **37** | 0% | 0% | 0% | 0% | 0% | 0% | 1% |
| **38** | 0% | 0% | 0% | 0% | 0% | 0% | 1% |
| **39** | 0% | 0% | 2% | 4% | 3% | 1% | 6% |
| **40** | 2% | 0% | 0% | 0% | 0% | 0% | 0% |
| **41** | 0% | 0% | 0% | 0% | 1% | 0% | 4% |
| **42** | 0% | 0% | 0% | 0% | 0% | 0% | 0% |
| **Total MRI-Positive Group** | 0% | 0% | 1% | 1% | 2% | 0% | 6% |

**Supplementary Table 5.** Subject-specific visual concordances with ground truth from MRI-negative cohort with conclusive SOZ

| **Subject** | **Visual Concordance** | | | | | | |
| --- | --- | --- | --- | --- | --- | --- | --- |
|  | **Increased GM** | **Decreased GM** | **Decreased FA** | **Increased MD** | **Decreased NDI** | **Increased FLAIR** | **NPC** |
| **1** | N | Y | Y | Y | Y | N | Y |
| **2** | Y | N | N | N | N | N | N |
| **3** | Y | N | Y | N | Y | Y | Y |
| **4** | N | N | N | N | Y | Y | Y |
| **5** | N | N | N | Y | Y | N | Y |
| **6** | N | N | N | N | N | N | N |
| **7** | N | N | N | N | N | N | N |
| **8** | N | N | N | N | N | N | N |
| **9** | Y | N | N | N | N | N | Y |
| **10** | N | N | N | N | N | Y | Y |
| **11** | N | N | N | N | N | N | N |
| **12** | N | N | N | N | N | N | N |
| **13** | N | Y | N | N | N | N | N |
| **14** | Y | N | N | Y | N | Y | Y |
| **15** | N | Y | Y | Y | Y | Y | Y |
| **16** | N | N | N | N | N | N | N |
| **17** | N | N | N | N | N | N | N |
| **18** | N | N | Y | Y | Y | N | Y |
| **Total**  **MRI-Negative Group** | 4/18 (22%) | 3/18 (17%) | 4/18 (22%) | 5/18 (28%) | 6/18 (33%) | 5/18 (28%) | 9/18 (50%) |

**Supplementary Table 6.** Subject-specific voxel-wise significant findings from MRI-negative cohort with inconclusive SEEG

| **Subject** | **Visual Concordance** | | | | | | |
| --- | --- | --- | --- | --- | --- | --- | --- |
|  | **Increased GM** | **Decreased GM** | **Decreased FA** | **Increased MD** | **Decreased NDI** | **Increased FLAIR** | **NPC** |
| **1** | N | N | N | N | N | N | N |
| **2** | N | N | N | N | N | N | N |
| **3** | N | N | N | N | Y | N | N |
| **4** | N | N | Y | N | N | Y | Y |
| **5** | N | N | N | N | N | N | N |
| **6** | N | N | N | N | N | N | N |
| **7** | N | N | N | N | N | N | N |
| **8** | N | N | N | N | Y | N | Y |
| **9** | N | N | N | N | N | N | N |
| **Total**  **MRI-Negative SEEG Inconclusive Group** | 0/9  (0%) | 0/9  (0%) | 1/9  (11%) | 0/9  (0%) | 2/9  (22%) | 1/9  (11%) | 2/9  (22%) |


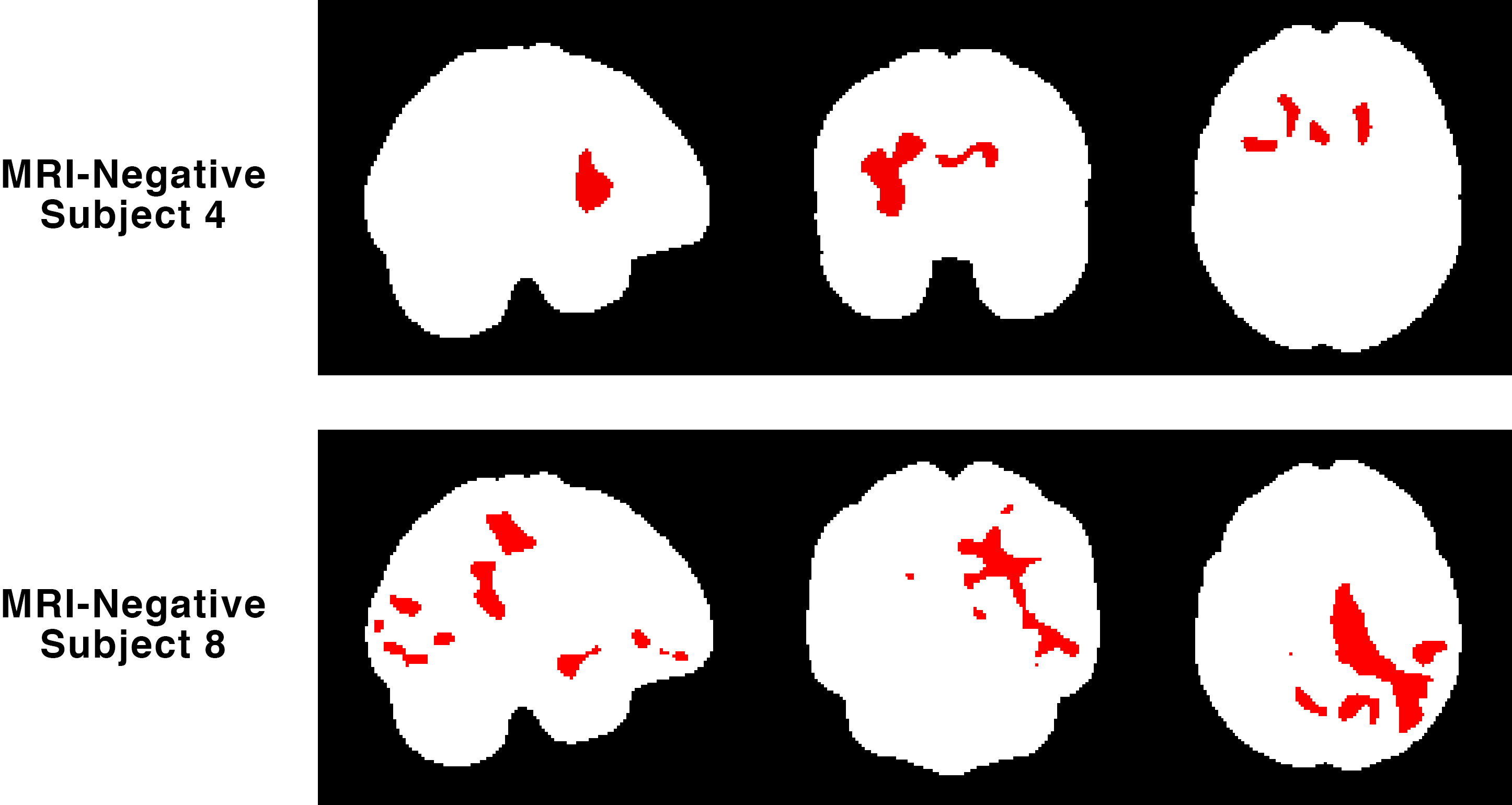


**Supplementary Figure 1.** Illustration of results from two MRI-negative, SOZ inconclusive subjects with significant findings from NPC analysis. The NPC analysis results are thresholded at p<0.05 (red), and overlaid on the brain mask.
